# Optimizing biomass estimates of savanna woodland at different spatial scales in the Brazilian Cerrado: Re-evaluating allometric equations and environmental influences

**DOI:** 10.1371/journal.pone.0196742

**Published:** 2018-08-01

**Authors:** Iris Roitman, Mercedes M. C. Bustamante, Ricardo F. Haidar, Julia Z. Shimbo, Guilherme C. Abdala, George Eiten, Christopher W. Fagg, Maria Cristina Felfili, Jeanine Maria Felfili, Tamiel K. B. Jacobson, Galiana S. Lindoso, Michael Keller, Eddie Lenza, Sabrina C. Miranda, José Roberto R. Pinto, Ariane A. Rodrigues, Wellington B. C. Delitti, Pedro Roitman, Jhames M. Sampaio

**Affiliations:** 1 Department of Ecology, Universidade de Brasília, Brasília, Distrito Federal, Brazil; 2 Brazilian Research Network on Global Climate Changes – Rede Clima, Ministério de Ciência, Tecnologia e Inovação, Brasília, Distrito Federal, Brazil; 3 Radis Project, Faculdade de Planaltina, Universidade de Brasília, Brasília, Distrito Federal, Brazil; 4 Universidade Estadual do Tocantins, Palmas, Tocantins, Brazil; 5 Instituto de Pesquisa Ambiental da Amazônia, Brasília, Distrito Federal, Brazil; 6 Instituto Avaliação - Pesquisa e Projetos Socioambientais, Brasília, Distrito Federal, Brazil; 7 Department of Botany, Universidade de Brasília, Brasília, Distrito Federal, Brazil; 8 Faculdade de Ceilândia, Universidade de Brasília, Brasília, Distrito Federal, Brazil; 9 Companhia de Saneamento do Distrito Federal, Brasília, Distrito Federal, Brazil; 10 Department of Forest Engineering, Universidade de Brasília, Brasília, Distrito Federal, Brazil; 11 Faculdade UnB Planaltina, Universidade de Brasília, Planaltina, Distrito Federal, Brazil; 12 Instituto Neotropical: Pesquisa e Conservação, Curitiba, Paraná, Brazil; 13 USDA Forest Service, International Institute of Tropical Forestry, Rio Piedras, Puerto Rico; 14 Jet Propulsion Lab, California Institute of Technology, Pasadena, California, United States of America; 15 Biology Department, Universidade do Estado de Mato Grosso, Campus Nova Xavantina, Nova Xavantina, Brazil; 16 Universidade Estadual de Goiás, Campus Palmeiras de Goiás, Palmeiras de Goiás, Brazil; 17 Departamento de Ecologia, Universidade de São Paulo, São Paulo, Brazil; 18 Department of Mathematics, Universidade de Brasília, Brasília, Brazil; 19 Department of Statistics, Universidade de Brasília, Brasília, Brazil; Chinese Academy of Forestry, CHINA

## Abstract

Cerrado is the second largest biome in South America and accounted for the second largest contribution to carbon emissions in Brazil for the last 10 years, mainly due to land-use changes. It comprises approximately 2 million km^2^ and is divided into 22 ecoregions, based on environmental conditions and vegetation. The most dominant vegetation type is cerrado *sensu stricto* (cerrado ss), a savanna woodland. Quantifying variation of biomass density of this vegetation is crucial for climate change mitigation policies. Integrating remote sensing data with adequate allometric equations and field-based data sets can provide large-scale estimates of biomass. We developed individual-tree aboveground biomass (AGB) allometric models to compare different regression techniques and explanatory variables. We applied the model with the strongest fit to a comprehensive ground-based data set (77 sites, 893 plots, and 95,484 trees) to describe AGB density variation of cerrado ss. We also investigated the influence of physiographic and climatological variables on AGB density; this analysis was restricted to 68 sites because eight sites could not be classified into a specific ecoregion, and one site had no soil texture data. In addition, we developed two models to estimate plot AGB density based on plot basal area. Our data show that for individual-tree AGB models a) log-log linear models provided better estimates than nonlinear power models; b) including species as a random effect improved model fit; c) diameter at 30 cm above ground was a reliable predictor for individual-tree AGB, and although height significantly improved model fit, species wood density did not. Mean tree AGB density in cerrado ss was 22.9 tons ha^-1^ (95% confidence interval = ± 2.2) and varied widely between ecoregions (8.8 to 42.2 tons ha^-1^), within ecoregions (e.g. 4.8 to 39.5 tons ha^-1^), and even within sites (24.3 to 69.9 tons ha^-1^). Biomass density tended to be higher in sites close to the Amazon. Ecoregion explained 42% of biomass variation between the 68 sites (P < 0.01) and shows strong potential as a parameter for classifying regional biomass variation in the Cerrado.

## Introduction

Cerrado, a wet seasonal savanna, is the second largest biome in South America. Between 2002 and 2010, the Cerrado accounted for the second largest contribution to net carbon emissions (1,845 Tg) in Brazil in the Land Use and Land-Use Change and Forest (LULUCF) sector [1]. Vegetation carbon stocks are much lower in the savanna than in Amazon forests (29 vs. 120 Mg C ha^-1^) [2]. However, land-use changes in the Cerrado are occurring much faster. In 2010, approximately 50% of its original habitat had been converted, mainly due to agricultural and livestock activities [3]. Mapping terrestrial carbon stocks is essential for climate change mitigation policies [4], and optimizing biomass and carbon estimates across a range of spatial scales is important to provide confidence in carbon markets and REDD+ projects [5]. Uncertainty in vegetation carbon stocks is high [6–8], especially in the Cerrado biome [7]; therefore, improving estimates of carbon stocks in the Cerrado is crucial to determine the impacts of land-use changes, understand their role in the global carbon balance, and support climate change mitigation policies.

The Cerrado covers approximately 2 million km^2^ and is divided into 22 ecoregions according to climate, geomorphology, soil, and vegetation [9]. As the Brazilian agricultural frontier moves toward the northwest of the Cerrado [10,11], regional estimates of biomass are needed to quantify the impact of regional patterns of deforestation on carbon balance. However, estimating biomass and carbon density of vegetation in the Cerrado is challenging because of its large latitudinal gradient and high environmental and structural variability. Besides variation across the many vegetation types [12,13], considerable variation exists within the same vegetation class [14].

The most dominant type of vegetation in the Cerrado is cerrado *sensu stricto* (cerrado ss), which consists of a continuous herbaceous grassy layer and a woody layer with 10%–60% canopy cover, where most trees are 3–5 m tall [15]. Its structure varies from sparse to dense woodland. Detecting fine-scale biomass variation of cerrado ss is a challenge for remote sensing carbon mapping. However, quantifying biomass density and disentangling the environmental aspects related to this variation should improve large-scale carbon stock estimates in the Cerrado. Integrating remote sensing data with adequate allometric equations and field-based data sets can provide large-scale estimates of biomass.

There are few allometric equations for cerrado ss vegetation. Error distributions for some of these equations have not been reported; therefore it is not possible to evaluate bias or determine whether regression analysis assumptions of homoscedasticity and normality of errors have been met [16,17]. Other equations result in negative biomass for small trees (diameter at 30 cm above ground ≤ 5 cm, and height ≤ 0.67 m) [18] or cover areas outside the Cerrado core region (e.g. Minas Gerais state) or transitional areas (e.g. Atlantic Forest) [19,20]. The most recent review on regional biomass variation in the Cerrado by Miranda et al. [21] made no progress toward the development of allometric equations. Furthermore, most sites were in the southern part of the biome [21].

In the present study, we developed and compared 12 allometric models to identify the regression techniques that provide the strongest fit and the most important explanatory variables to estimate individual-tree AGB for cerrado ss. We focused on the following questions: a) Do log-log linear models provide better estimates than power models? b) In multispecies models, does including a species random effect improve model fit? c) Is diameter a good predictor of individual-tree AGB? d) Does including height and species wood density improve model fit?

We used the individual-tree AGB model with the strongest fit to estimate AGB density of cerrado ss in 77 sites and assess regional variation within the Cerrado biome. We also investigated the influence of the following physiographic and climatological variables on AGB variation: ecoregion, soil texture, and climatic factors (climatological water deficit and environmental stress). This analysis was restricted to 68 sites because eight sites could not be classified into a specific ecoregion, and one site had no soil texture data.

Improving large-scale carbon estimates in the Cerrado requires a large number of ground-based data sets. Individual-tree data are scarce and difficult to obtain, but plot data are more common in the literature. Therefore, we used a comprehensive individual-tree data set of 893 plots (95,484 trees) in 77 sites to develop two models to estimate plot AGB density based on plot basal area.

### Nonlinear regression x log-log transformed data

Many allometric relationships in nature can be described by power functions (or power law). The classic example is Kleiber’s law, in which basal metabolic rate is expressed as a function of body mass (y = ax^3/4^) [22]. West et al. [23] developed a quantitative model to explain the origin and universality of the power law based on three assumptions: the nutrient transport network follows a fractal pattern, the smallest branch is size-invariant, and the energy required to distribute resources is minimized. West et al. later proposed a general allometry model for vascular plants in which biomass scales with diameter (y = ax^8/3^) [24]. Muller-Landau et al. [25] criticized the generalization of the metabolic scaling theory and suggested that scaling also depends on asymmetric competition and availability of resources, such as light. A single constant coefficient for the scaling rule has been refuted [25], but the structure of power-law models is widely used to develop biomass allometric models [26]:

$$y=ax^{b}+\varepsilon ,\;\varepsilon \;\sim \;N(0,\sigma^{2})$$

where *y* = response variable, *x* = explanatory variable, *a* and *b* are model parameters, and *ε* = error, which is assumed to be normally distributed with zero mean.

In most statistical packages, the default nonlinear regression (NLR) technique (least-squares fit) assumes homogeneity of errors [27]. However, because this assumption is often violated for allometry data [4], the use of NLR power models may result in substantial bias [27,28]. Power models can be directly converted to linear form by log-transformation of response and explanatory variables (log-log transformation):

$$ln(y)=ln(a)+b\cdot ln(x)\;+\;\varepsilon ,\;\varepsilon \;\sim \;N(0,\sigma^{2}).$$

It is convenient to define *α* = *b*, *β* = ln(*a*), and rewrite the equation above as

$$ln(y)=\alpha \cdot ln(x)\;+\beta +\;\varepsilon ,\;\varepsilon \;\sim \;N(0,\sigma^{2})$$

where α and β are model parameters.

Log-log transformation may result in homoscedastic errors [27,28], motivating the widespread use of log-log transformation followed by linear regression (LR) in biomass allometric models. A theoretical reason for using log-log transformation is that allometry (how the size of one body part changes with respect to another) measures proportional relationships, not absolute relationships. Thus, log-log transformation allows proportional relationships to be readily quantified, unlike the original arithmetic data [29]. Many allometric relationships are multiplicative by nature, and log-log-transformation is useful because accounting for proportional variation is most important [30]. Some argue that log-log LR models can be biased and misleading [31–34], but others advocate their use as a better approach [27, 35–38].

Xiao et al. developed a simple method to compare NLR and log-log LR based on the error distribution [35]. NLR assumes that the error is normally distributed and additive on the arithmetic scale [29], whereas LR assumes that the error is normally distributed and additive on the logarithmic scale [30], which corresponds to lognormally distributed and multiplicative on the arithmetic scale [35]. We used this method to compare NLR and log-log LR methods in fitting AGB models to our cerrado ss data.

## Materials and methods

**Fig 1:**
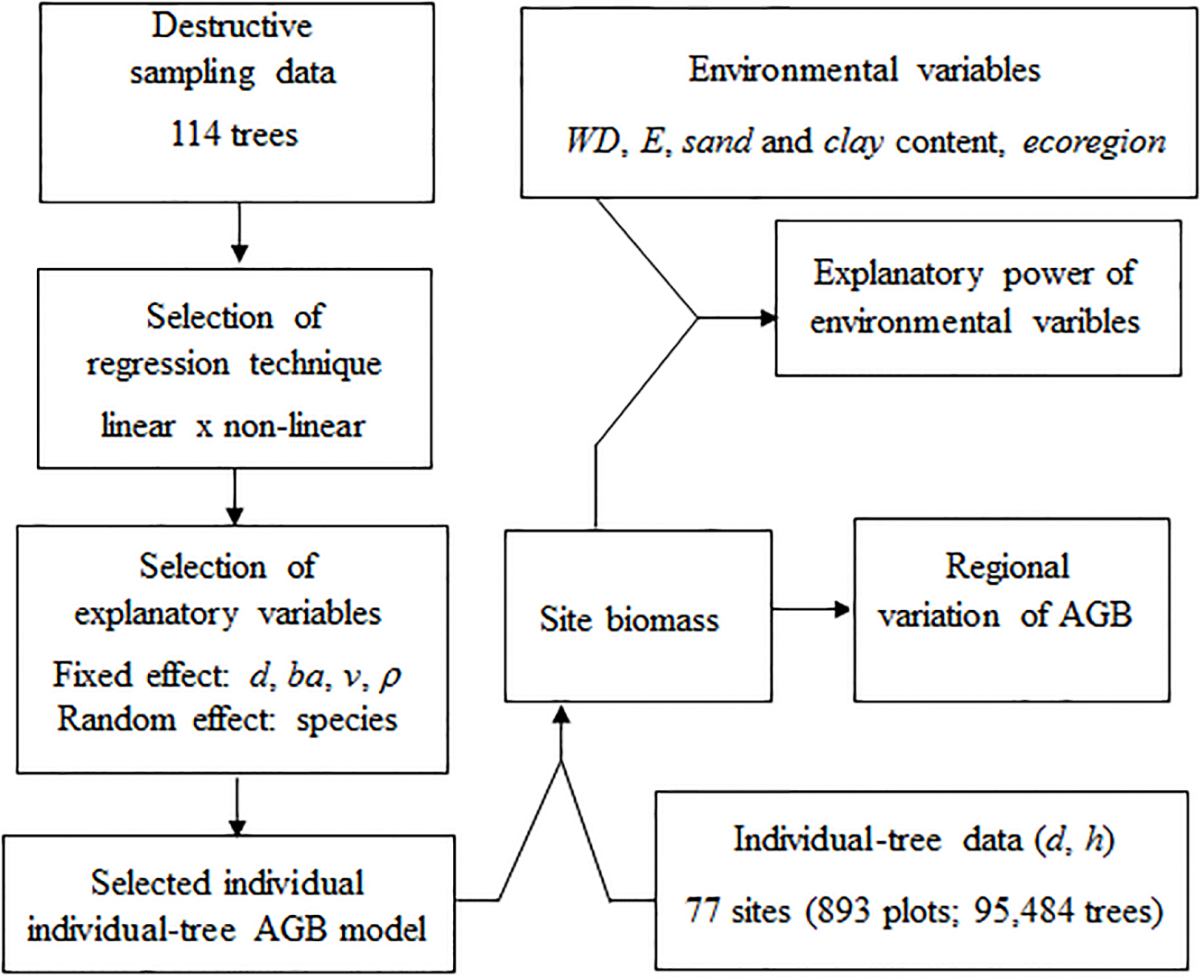
Methodological steps for developing an individual-tree aboveground biomass (AGB) model for cerrado *sensu stricto* in the cerrado, determining regional variation of tree AGB density, and evaluating environmental factors as explanatory variables. *d* = diameter, *ba* = basal area, *v* = volume, *h* = height, *ρ* = species wood density; *WD* = climatological water deficit; and *E* = environmental stress.

The study was divided in a series of steps: a) evaluating regression techniques and variables to identify the individual-tree AGB model with the strongest fit; b) using the selected model to estimate and determine biomass variation of cerrado ss in the Cerrado; and c) determining the influence of explanatory variables on this variation (Fig 1). We also developed models to estimate plot AGB density based on plot basal area data.

### Tree aboveground biomass allometric models

#### Destructive sampling

We used destructive sampling data collected by Prof. George Eiten’s team between 1982 and 1990. George Eiten (1923–2012) was Professor of the Botany Department of the University of Brasília, from 1971 to 1993. A model published in Abdala et al. [16] was based on 112 trees of this same data set. Trees were collected from a cerrado ss, located along the outer edge (3.5×150 m) of the Brasília Botanical Garden (BBG) (15°54'53''S, 47°49'33''W; altitude, approximately 1165 m). Although trees were harvested outside BBG, the vegetation was well preserved and retained the structural characteristics of this vegetation type. The terrain is flat, and the soil is red Oxisol with medium to sandy texture.

The sampling efforts comprised species common to cerrado ss vegetation [39]. Two field campaigns were carried out per year at the beginning and end of the rainy season (total of 16 field campaigns) to avoid dry season deciduousness of most sampled species. Trees were selected based on the following criteria: species, size variation within species, and tree integrity. Before harvest, tree diameter at 30 cm above ground (d) and total height (h) were measured. Large tarpaulins were placed on the ground to collect sawdust and splinters from cutting or sawing. Trees were harvested from top to bottom, in the following order: new leaves and current-year branches, old leaves, thin branches (≤ 2 cm diameter), thick branches (> 2 cm diameter), and trunk.

The harvested material was separated into compartments (trunk slices, thick branches, thin branches, and leaves) and then carefully placed into thick plastic bags that were previously marked and weighed. The samples were transported to the lab, where fresh weight was immediately recorded. After oven-drying the samples (65°C for leaves, and 100°C for other compartments) to constant weight, dry weight was recorded.

The final destructive sampling set (S1 Table) consisted of 114 trees from eight species very common in cerrado ss [39]: *Byrsonima coccolobifolia* Kunth, (n = 20) *Byrsonima verbascifolia* (L.) DC. (n = 15), *Connarus suberosus* var. fulvus (Planch.) Forero (n = 16), *Dalbergia miscolobium* Benth. (n = 15), *Palicourea rigida* Kunth (n = 20), *Piptocarpha rotundifolia* (Less.) Baker (n = 10), *Pterodon pubescens* (Benth.) Benth. (n = 4), and *Qualea grandiflora* Mart. (n = 14). Despite high beta-diversity in the Cerrado biome, a few dominant species (oligarchic species) often account for most of the total denisity in many physiognomies [39–41].

**Fig 2:**
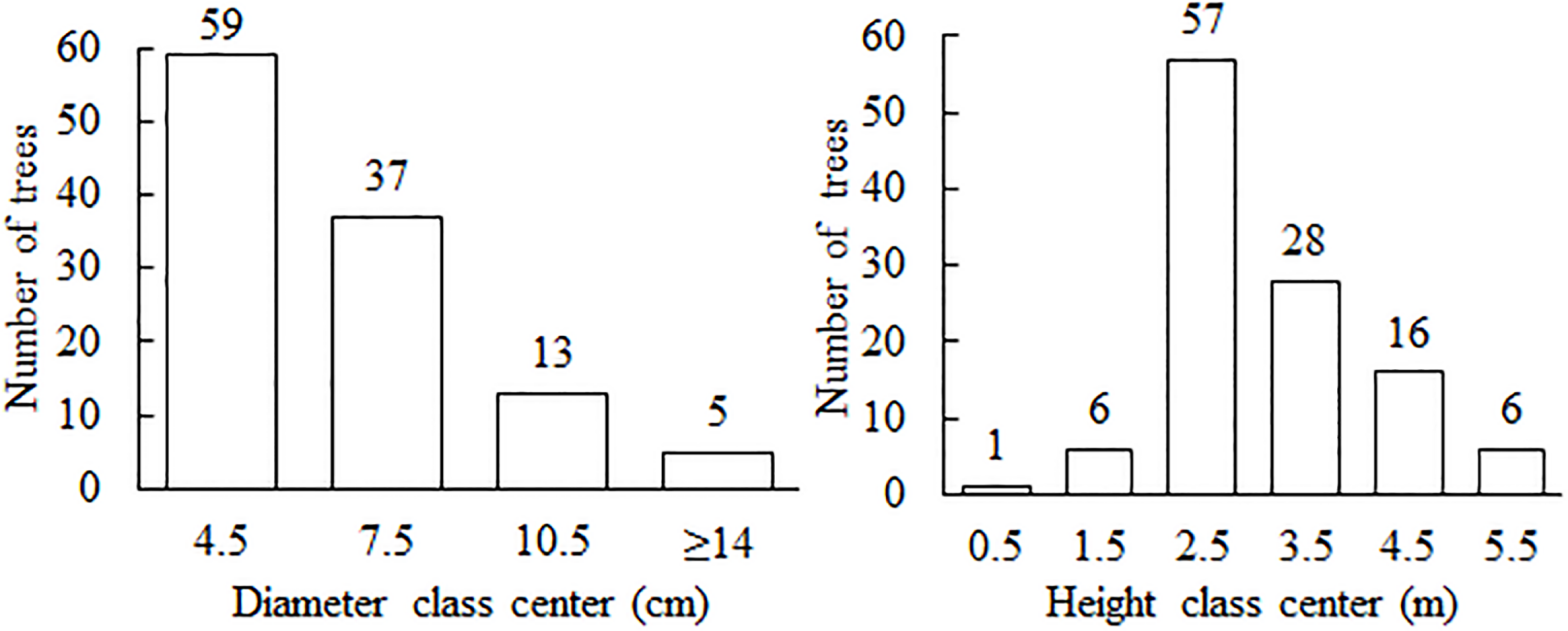
Diameter and height distributions of trees sampled outside Brasília Botanical Garden in Brazil used to develop allometric biomass equations.

Tree diameter ranged from 2.75 to 15.5 cm, and the distribution followed a reverse-J pattern, which is common to well-preserved cerrado ss. Most trees (74%) had height between 1 and 3 m (Fig 2). Species wood density values were obtained from the literature [42] and ranged from 0.42 g cm^-3^ (*P*. *rotundifolia*) to 0.73 g cm^-3^ (*P*. *pubescens*) (S1 Table).

#### Individual-tree aboveground biomass model construction

We developed 12 individual-tree AGB allometric models (Table 1) in order to a) compare NLR and LR techniques to fit the simple power-law model; b) investigate whether including species as a random effect improves the model fit; and c) evaluate the following explanatory variables: diameter (*d*), basal area (*ba*), trunk cylindrical volume (*v*), and species wood density (ρ) (Table 1).

**Table 1:** Allometric models to estimate individual-tree aboveground biomass of cerrado *sensu stricto*, based on different explanatory variables (diameter, basal area, volume, and wood density) and species as random effect.

Model	Type	X	Model structure
1	LR	*d*	*ln* (*y*_*i*_) = *α* · *ln*(*x*_*i*_) + *β* + *ε*_*i*_, *ε*_*i*_ ~ *N*(0, *σ*^2^)
2		*v*	
3	NLR	*d*	*y*_*i*_ = *α*(*x*_*i*_)^*β*^ + *ε*_*i*_, *ε*_*i*_ ~ *N*(0, *σ*^2^)
4		*v*	
5	GLM	*d*	*ln* (*y*_*i*_) = *α* · *ln*(*x*_*i*_) + *β* + *ε*_*i*_, *ε*_*i*_ ~ *N*(0, *σ*^2^)
6		*ba*	
7		*v*	
8		*v*ρ	
9	GLMM	*d*	*ln* (*y*_*ij*_) = *α* · *ln*(*x*_*ij*_) + *β* + *u*_*j*_ + *ε*_*ij*_, *ε*_*ij*_ ~ *N*(0, *σ*^2^)
10		*ba*	
11		*v*	
12		*v*ρ	

LR = linear regression, NLR = nonlinear regression, GLM = generalized linear model, GLMM = generalized linear mixed-effect model, *d* = diameter (cm), *ba* = basal area (cm^2^), *v* = volume (dm^3^), *v*ρ = volume (dm^3^) · species wood density (g dm^-3^), *y*_*i*_ = aboveground biomass (g) of tree *i*, *x*_*i*_ = explanatory variable of tree *i*, *ε*_*i*_ = error associated with tree *i*, *y*_*ij*_ = aboveground biomass (g|) of tree *i* from species *j*, *x*_*ij*_ = explanatory variable of tree *i* from species *j*, *u*_*j*_ = random-effect parameter generated by species effect, and *ε*_*ij*_ = error associated with tree *i* from species *j*.

To identify the regression technique that provides the strongest fit, we compared the LR models (models 1 and 2) against their corresponding NLR models (models 3 and 4, respectively). To determine whether including species as a random effect improves model fit, we used generalized linear models (GLMs) with Gaussian distribution (models 5, 6, 7, 8), which are equivalent to the log-log linear models, to enable direct comparison with generalized linear mixed-effect models (GLMMs; models 9, 10, 11, 12, respectively). To evaluate explanatory variables, we compared models with the same regression methods.

All simulations and analyses to compare LR and NLR models were run in R version 2.15.3 [43], with packages “nlrwr” [44] and “boot” [45,46]. All remaining procedures for model simulation and analysis were performed in R version 3.2.4 revised [47], packages MuMIn [48] and lme4 [49]. For GLMs and GLMMs, we used maximum likelihood fit and Gaussian error family.

Back-transformation of log-log LR models to the power-law form requires a correction factor that accounts for skewness of the distribution of *y*, based on the residual standard error (σ) [50–52].

Linear form: *ln* (*y*) = *α ln* (*x*) + *β*

Power-law form: *y* = *e*^*β*^*x*^*α*^

CF=e(σ2)

$$\sigma =\sqrt{\frac{\sum {\left[\text{ln}\left(y_{i}\right)-\text{ln}\left({\hat{y}}_{i}\right)\right]}^{2}}{N-k}}$$

where *CF* = correction factor, *σ* = residual standard error, *N* = total number of sampled trees, *y*_*i*_ = *i*^th^ observed biomass, ${\hat{y}}_{i}=i^{\text{th}}$ estimated biomass, and *k* = number of parameters.

#### Individual-tree aboveground biomass model analysis

We compared LR and NLR models with the method proposed by Xiao et al. [36–38]. The NLR technique is suitable for data with additive, homoscedastic, normal error, whereas log-log LR performs better for data with multiplicative, heteroscedastic, lognormal error (see [34] for a detailed description of the method).

All models were analyzed in terms of error distribution (homoscedasticity and normality), uncertainty of model parameters α and β (standard error, percent relative standard error, and confidence intervals) [8], residual standard error, coefficient of variation (CV) [4], P-value, and Akaike information criterion (AIC). The analysis also included the coefficient of determination (R^2^) for simple LR models, McFadden’s pseudo R^2^ for GLMs, and marginal and conditional R^2^ for GLMMs [40]. Marginal R^2^ (R^2^m) represents the variance explained by fixed factors, and conditional R^2^ (R^2^c) represents the variance explained by both fixed and random-effect factors.

CV=100·(σy-)

where CV = coefficient of variation, σ = residual standard error, and $\overset{-}{y}$ = mean of the response variable y.

The model with the strongest fit was back-transformed, and we assessed its performance with an independent validation set (S2 Table), used by Delitti et al. [17].

### Plot biomass density models

#### Construction of plot biomass density models

We developed two mixed-effect models (with site as random effect) to estimate plot AGB density from plot basal area. We used a comprehensive ground-based data set (diameter and height), consisting of 893 plots within 77 cerrado ss sites. This data set covers a wide latitudinal and longitudinal range (6°4'17.22''S to 19°10'53.184''S; 42°29'30.84''W to 56°13'30''W). The plots were 20 × 50 m (0.1 ha), except for those in site 77, which were 20 × 20 m. All inventories included trees with base diameter ≥ 5 cm (at 30 cm above ground). Additional details on the data set are presented in S3 Table.

First, we estimated plot basal area (explanatory variable) for 893 plots. Then we estimated individual-tree AGB with models 10 and 11 to calculate plot AGB density (response variable) (S4 Table) and to develop models 14 and 13, respectively, using maximum likelihood fit with Gaussian distribution:

$$ln(y_{ps})=\alpha \;\cdot ln(x_{ps})+\beta +\;u_{s}+\;\varepsilon_{ps},\;{\;\varepsilon }_{ps\;}\sim \;N(0,\sigma^{2})$$

where *y*_*ps*_ = aboveground biomass density (ton ha^-1^) of plot *p* from site *s*, *x*_*ps*_ = plot basal area (m^2^ ha^-1^) of plot *p* from site *s*, *u*_*s*_ = random-effect parameter generated by site effect, and *ε*_*ps*_ = error associated with plot *p* from site *s*.

#### Analysis of plot biomass density models

Models were evaluated in terms of marginal and conditional R^2^ [53], P-value, CV, and AIC. Assumptions of normality and homoscedasticity of errors were checked. All simulations and analyses were performed in R (R Core Team 2017) with packages MuMin [48] and lme4 [49].

### Variation in tree aboveground biomass density of cerrado *sensu stricto*

We used the selected model to estimate tree AGB density in 77 of the cerrado sites. For each of the sites, we calculated AGB density confidence intervals based on variability between plots. Significant differences in biomass density between sites were determined with the Kruskal-Wallis test (P < 0.05). We also applied hierarchical clustering (using Euclidean distance matrix computation) to separate groups based on biomass densities with package Mass [54] in R [47].

### Factors influencing plot aboveground biomass density variation of cerrado *sensu stricto*

We used LR and GLMMs to determine the effect of the following variables on tree AGB variation: maximum climatological water deficit (*CWD*), environmental stress (*E*) [4], soil (sand and clay content) [55], and ecoregion [9].

*CWD* is the sum of the difference between monthly rainfall (*P*_*i*_) and monthly evapotranspiration (*ET*_*i*_) when this difference is negative (water deficit): $CWD=\sum_{i=1}^{12}Min(0,{\;P}_{i}-{ET}_{i})$ [4]. Environmental stress is based on *CWD*, seasonal temperature (*TS*), and seasonal precipitation: *E* = (0.178 · *TS* − 0.938 · *CWD* − 6.61 · *PS*) · 10^−3^. Chave et al. provided *CWD* and *E* on a global gridded layer at 2.5-arcsec resolution [4] (available at http://chave.ups-tlse.fr/pantropical_allometry.htm). Sand content (50–2000 μm mass fraction (%) at 0–30 cm depth) and clay content (0–2 μm mass fraction (%) at 0–30 cm depth) was obtained from a 250-m soil grid (SoilGrids) [55].

We used the classification of Cerrado ecoregions (1:250.000) [9] derived from the Land System Classification [56] and followed the criteria of Bailey [57] and Dinerstein [58] based on six controlling factors, in order of importance: geomorphology, geology, soil, precipitation, vegetation classification, and presence/absence of key plant taxa. They used three families (Bromeliaceae, Loranthaceae and Viscaceae) and eight genera: *Cyrtopodium* (Orchidaceae), *Habenaria* (Orchidaceae), *Jacaranda* (Bignoniaceae), *Miconia* (Melastomataceae), *Mimosa* (Leguminosae), *Tabebuia* (Bignoniaceae), *Solanum* (Solanaceae), and *Vernonia* (Asteraceae). They first classified the Cerrado into 43 geomorphological units, which was reduced to 29 units by including geology, soil, and precipitation, and finally to 22 ecoregions by including vegetation class and key taxa. We restricted this analysis to 68 sites in 13 ecoregions because eight sites could not be classified into a specific ecoregion, and one site had no soil texture data.

## Results

### Tree aboveground biomass allometric models

#### Log-log linear models provided better estimates than power models

The NLR models (models 3 and 4) had heteroscedastic and non-normal errors, whereas the LR models (models 1 and 2) had homoscedastic and normal errors (Figures A–D in S1 File). The Δ*m* AIC_C_ between LR and NLR models was much greater than |2|, supporting the assumption of multiplicative lognormal error in models based on *d* and *v* (Table 2) and demonstrating that log-log LR models were more appropriate for our data set.

**Table 2:** Comparison of log-log linear and non-linear models for individual-tree aboveground biomass of cerrado *sensu stricto* in Brazil.

Model	3	1	4	2
Model structure	*y* = *a* · *d*^*b*^	*ln* (*y*) = *α* · *ln* (*d*) + *β*	*y* = *a* × *v*^*b*^	*ln* (*y*) = *α* · *ln* (*v*) + *β*
*a* (95% CIL)	82.41 (37.32, 167.32)		469.95 (288.89, 717.67)	
PRSE (%)	43.8		24.08	
*b* (95% CIL)	2.10 (1.82, 2.41)		0.97 (0.86,1.09)	
PRSE (%)	8.29		6.42	
*α* (95% CIL)		2.88 (2.67, 3.09)		0.99 (0.94, 1.05)
PRSE (%)		3.72		2.78
*β* (95% CIL)		2.44 (2.05, 2.84)		5.96 (5.84, 6.07)
PRSE (%)		8.18		0.98
CF		1.267		1.199
$R_{Adj}^{2}$		0.87		0.92
AIC	2265.119	156.739	2202.059	96.189
P-value		< 2.^2e-16^		< 2.2^e-16^
CV (%)	96.6	6.2	73.45	4.7
*m*AICc	2265.338	1909.912	2202.28	1849.36
Δ AIC_C_	355.4267		352.9197	

*a*, *b*, *α*, and *β* are model parameters, *d* = diameter (cm), *v* = volume (dm^3^), *y* = individual-tree aboveground biomass (g), PRSE = percent relative standard error of model parameters, $R_{Adj}^{2}$ = adjusted coefficient of determination, AIC = Akaike information criterion, CIL = confidence interval limits, CV = coefficient of variation, *mAICc* = second order variant of AIC.

#### Including species as random effect improved model fit

All GLMs and GLMMs had homoscedastic and normal errors (Figures E–L in S1 File). With the same explanatory variables, all GLMMs showed better performance than their corresponding GLMs, with the difference in AIC > |2| (Table 3).

**Table 3:** Comparison of generalized linear models (GLMs) and generalized linear mixed-effect models (GLMMs) to estimate individual-tree aboveground biomass, based on different explanatory variables (*x*): diameter (*d*), basal area (*ba*), volume (*v*), and volume · wood density (*v*ρ).

GLM		*α*			*β*			R^2^pseudo		AIC	CV (%)	CF
Model	*x*	coef. (95% CIL)	SE	PRSE (%)	coef. (95% CIL)	SE	PRSE (%)					
5	*d*	2.884 (2.68, 3.09)	0.107	3.7	2.444 (2.05, 2.84)	0.200	2.4	0.87		156.74	6.2	1.27
6	*ba*	1.442 (1.34, 1.55)	0.054	3.7	2.792 (2.43, 3.16)	0.187	2.8	0.87		156.74	6.2	1.27
7	*v*	0.997 (0.94, 1.05)	0.028	2.8	5.957 (5.84, 6.07)	0.059	6.8	0.92		96.19	4.7	1.20
8	*v*ρ	0.951 (0.90, 1.00)	0.026	2.8	0.073 (-0.34, 0.49)	0.213	0.1	0.92		95.92	4.7	1.20
GLMM		*α*			*β*			R^2^m	R^2^c	AIC	CV (%)	CF
Model	*x*	coef. (95% CIL)	SE	PRSE (%)	coef. (95% CIL)	SE	PRSE (%)					
9	*d*	2.776 (2.58, 2.97)	0.026	1.0	2.685 (2.27, 3.11)	0.208	2.7	0.85	0.89	141.00	6.2	1.22
10	*ba*	1.388 (1.29, 1.49)	0.050	3.6	3.020 (2.63, 3.43)	0.198	3.0	0.85	0.89	141.00	6.2	1.22
11	*v*	0.975 (0.92, 1.03)	0.026	2.7	6.014 (5.84, 6.20)	0.084	6.0	0.92	0.94	81.80	4.7	1.17
12	*v*ρ	0.963 (0.91, 1.02)	0.036	2.7	-0.020 (-0.46, 0.41)	0.220	-0.02	0.92	0.94	80.90	4.7	1.17

For all models, P < 0.001, *x* = explanatory variable, α and β are model parameters, coef. = coefficient, CIL = confidence interval limits, SE = standard error of the parameter, PRSE = percent relative standard error of the parameter, R^2^pseudo = pseudo coefficient of determination, R^2^m = marginal coefficient of determination, R^2^c = conditional coefficient of determination, AIC = Akaike information criterion, CV = coefficient of variation, and CF = correction factor.

#### Diameter and basal area were good predictors of individual-tree aboveground biomass, and including height improved model fit

All log-log linear models (LRs, GLMs, and GLMMs) based on diameter or basal area (models 1, 5, 6, 9, and 10) had low CVs (6.2%), demonstrating that diameter or basal area alone were good predictors of individual-tree AGB. For all model types, models based on *v* performed better than the corresponding models based on *d* or *ba* (Tables 2 and 3). Therefore, including *h* (as cylindrical volume) significantly improved model fit.

#### Including wood density did not improve model fit

Including wood density did not improve the fit for GLMs or GLMMs. Models 8 and 9 had the same R^2^m, R^2^c, and CV, and the absolute difference between AICs was > 2. Similarly, models 11 and 12 had the same R^2^m, R^2^c, and CV, and AICs did not differ significantly (Table 3). Considering the principle of parsimony, we suggest using model 11 to estimate tree AGB for cerrado ss. Model 11 was back-transformed (*y* = (409.047 · *v*^0.976^) · 1.17) and validated with an independent data set. The results demonstrated good performance, with a lower CV for the validation data set than for the training data set (Table 4).

**Table 4:** Performance of model 11, back-transformed to its power-law form (*y* = (409.047 · *v*^0.976^) · 1.17), using the training data set (present study) and an independent validation set from Delitti et al. [17].

Data set	N	SE (g)	CV (%)
Training data set	114	3,728	73.6
Validation data set	60	6,668	43.2

SE = standard error, CV = coefficient of variation, *y* = tree aboveground biomass (g), and *v* = tree volume (dm^3^).

### Tree aboveground plot biomass allometric models

Models 13 and 14 both had homoscedastic and normal errors (Figures M and N in S1 File), high R^2^m, and low CV (Table 5). Model 14 had higher R^2^m, lower CV, and lower AIC (Table 5).

**Table 5:** Evaluation of models 13 and 14 to estimate tree aboveground plot biomass density of cerrado *sensu stricto*.

	Model 13	Model 14
α (95% CIL)	1.197 (1.168, 1.227)	1.22043 (1.179, 1.25)
PRSE (%)	1.25	1.10
β (95% CIL)	0.245 (0.166, 0.323)	0.119 (0.050, 0.188)
PRSE (%)	16.30	29.22
R^2^m	0.88	0.91
R^2^c	0.95	0.96
P	< 2.2e-^16^	< 2.2e-^16^
CV (%)	5.34	4.92
AIC	-498.7	-680.4
CF	1.08	1.07
Power-law form	*y*_*plot*_ = (1.277 · *x*_*plot*_^1.197^) · 1.08	*y*_*plot*_ = (1.173 · *x*_*plot*_^1.220^) · 1.07

α and β are model parameters, PRSE = percent relative standard error of the parameters, CIL = confidence interval limits, R^2^m = marginal determination coefficient, R^2^c = conditional determination coefficient, AIC = Akaike information criterion, CV = coefficient of variation, CF = correction factor, *y*_*plot*_ = aboveground plot biomass (ton ha ^-1^), and *x*_*plot*_ = plot basal area (m^2^ ha^-1^).

### Biomass variation in 77 cerrado *sensu stricto* sites

**Fig 3:**
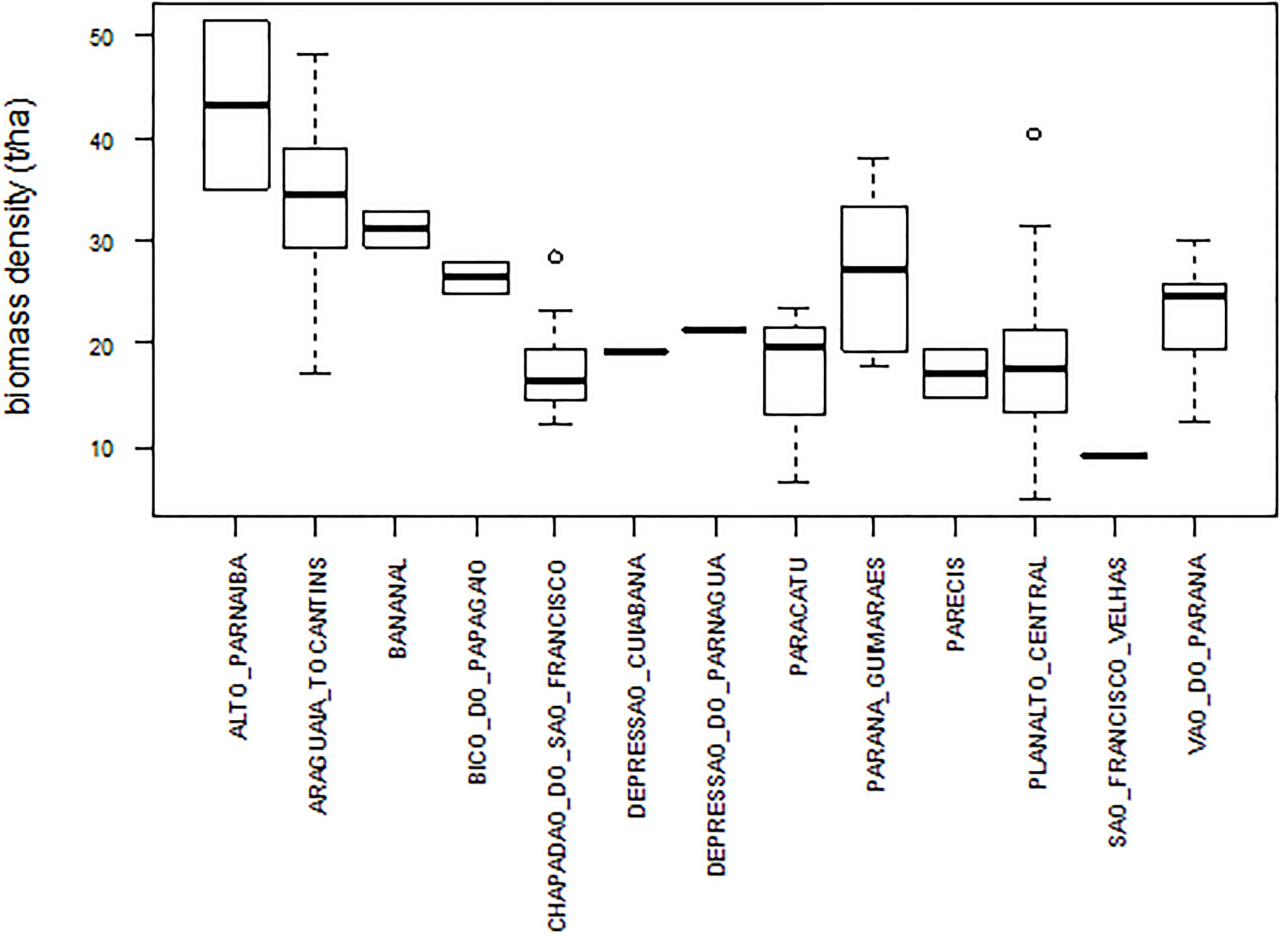
Tree aboveground biomass density of cerrado *sensu stricto* in 13 cerrado ecoregions, estimated with model 11.

**Fig 4:**
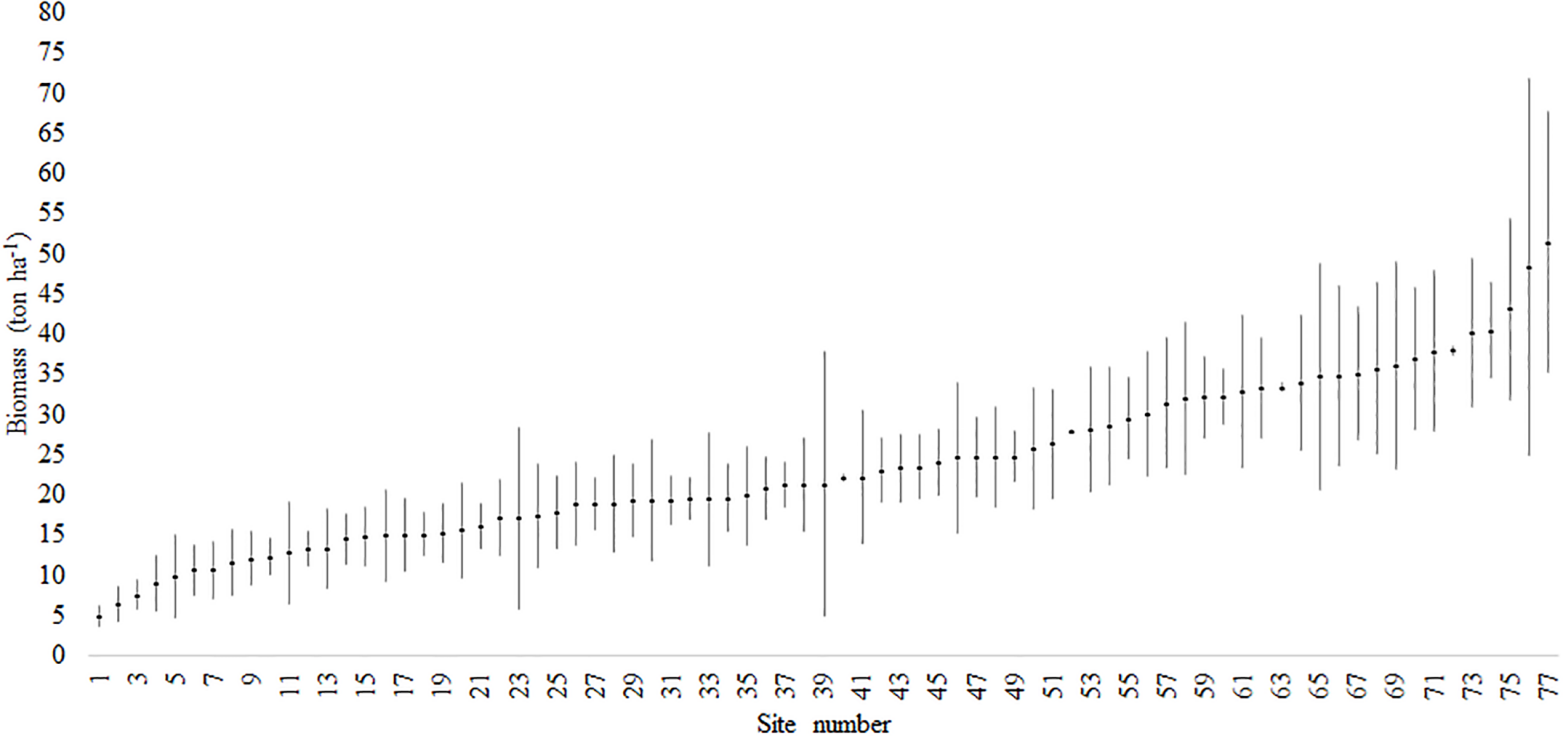
Tree aboveground biomass density and confidence interval of 77 cerrado *sensu stricto* sites, estimated with model 11.

Mean AGB of the 77 sites was 22.9 tons ha^-1^ (95% confidence interval = ± 2.2), with normal distribution (Shapiro–Wilk test: W = 0.97, P > 0.09) (Figure T in S1 File). AGB varied from 4.8 to 50.2 tons ha^-1^ with high CV (42.9%). Variation between sites was significant (P < 0.05) (S5 Table). Across ecoregions, mean AGB ranged from 8.8 tons ha^-1^ (São Francisco das Velhas) to 42.2 tons ha^-1^ (Alto Parnaíba), with high variation within ecoregions (e.g. 4.8 to 39.5 tons ha^-1^ in Planalto Central) (Fig 3). In many cases, within-site variation was also high, with large confidence intervals (e.g. 24.3 to 69.9 tons ha^-1^ in site 76) (Fig 4, Figure T in S1 File, S3 Table). Hierarchical clustering divided the sites into two categories: biomass density ≤ 24.1 tons ha^-1^ (sites 1–46); and biomass density ≥ 24.1 tons ha^-1^ (sites 47–77), except for site 48 (24.2 ton ha^-1^) that fell into the first category (Figure V in S1 File).

**Fig 5:**
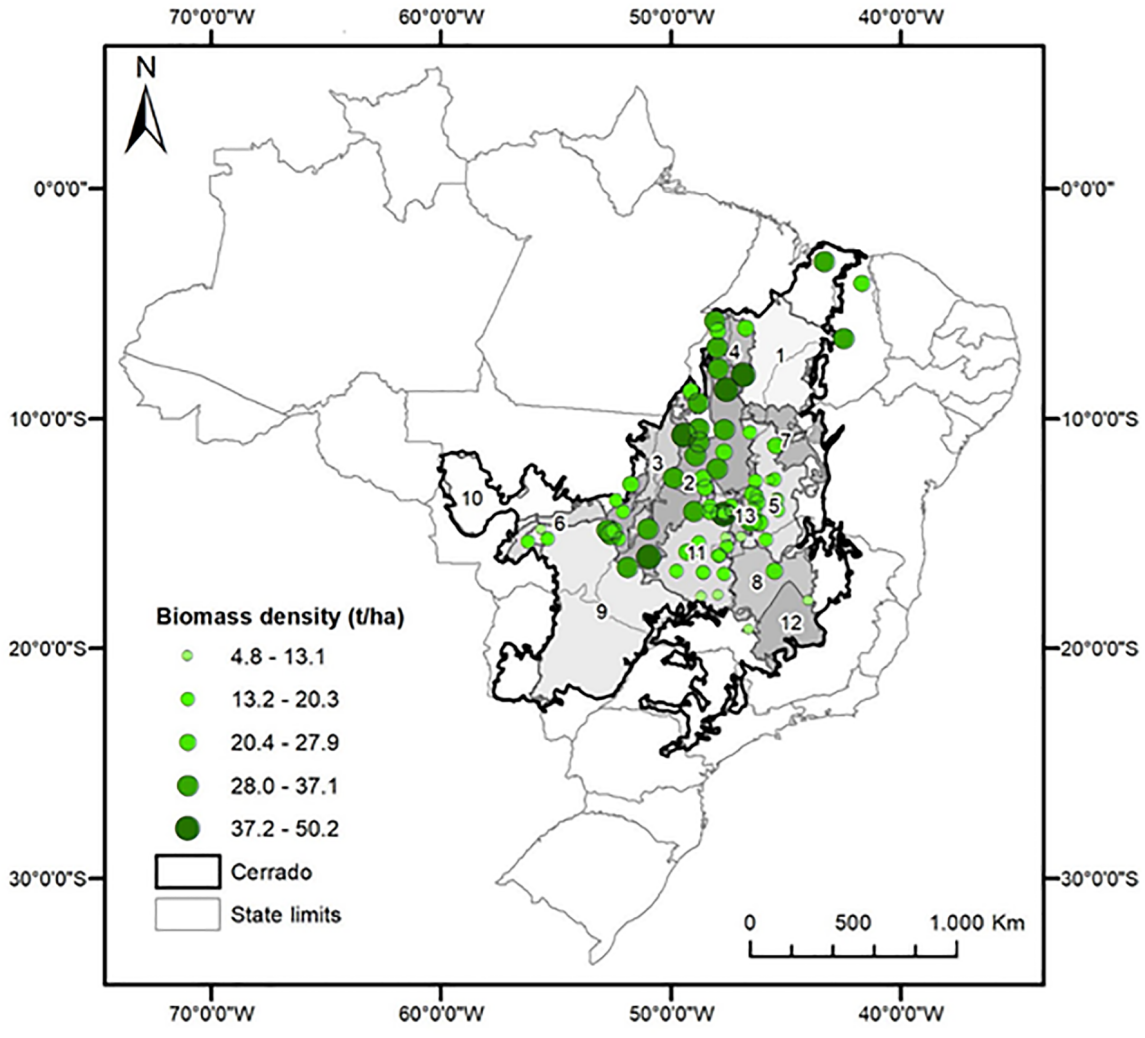
Distribution of tree aboveground biomass density of cerrado *sensu stricto* vegetation in cerrado (estimated with model 11), using individual-tree data from 77 sites. Numbers indicate ecoregions: 1 = Alto Paranaíba, 2 = Araguaia Tocantins, 3 = Bananal, 4 = Bico do Papagaio, 5 = Chapadão do São Francisco, 6 = Depressão Cuiabana, 7 = Depressão do Parnaguá, 8 = Paracatu, 9 = Paraná Guimarães, 10 = Parecis, 11 = Planalto Central, 12 = São Francisco Velhas, 13 = Vão do Paranã. Delimitation of Cerrado biome and ecoregions was obtained from IBGE [59] and Arruda et al. [9], respectively.

Although the spatial distribution of AGB density varied widely, even between nearby sites, there is a regional pattern in which biomass density tended to be higher in eastern sites, closer to the Amazon (Fig 5).

### Environmental variables

When examined individually with simple LR, ecoregion explained 42% of AGB variation between 68 sites (P < 0.05); sand and clay explained 11.5% and 7.4% of the variation, respectively (P < 0.05) (Table 6). All models had normal and homoscedastic errors (Figures O–S in S1 File).

**Table 6:** Effect of environmental factors on tree aboveground biomass density of 68 cerrado *sensu stricto* sites in Brazil, using LR models.

Model	Explanatory variables	$R_{Adj}^{2.}$	P-value	CV (%)	AIC
15	CWD	0.028	0.093	43.79	506.47
16	E	-0.01	0.533	43.79	509.01
17	Sand	0.115	0.002	41.78	500.09
18	Clay	0.074	0.014	42.72	503.12
19	Ecoregion	0.424	1.2E-05	33.71	480.51

CWD = climatological water deficit, E = environmental stress, $R_{Adj}^{2}$ = adjusted determination coefficient, CV = coefficient of variation, and AIC = Akaike information criterion.

When considering ecoregion as random effect, *clay* + *sand* × CWD explained 15% of AGB variation (R^2^m = 0.15, P = 0.014, CV = 30.2%). Although significant effects were observed for clay (P = 0.020) and *sand* x *CWD* (P = 0.004), the variation was explained primarily by random (ecoregion) and fixed-effect factors combined (R^2^c = 0.53).

## Discussion

### Tree aboveground allometric models

#### Log-log linear models provided better estimates of tree aboveground biomass

Our data corroborate previous studies [27,35,38,60] that support the use of log-log LR over NLR to estimate tree AGB. In the theoretical model (y = ax^b^) of West et al. [24], the exponent b = 2.67. Our nonlinear diameter-based model (model 3) had a much lower exponent (2.10), but when back-transformed to power-law form, exponents of diameter-based log-log LR models were closer to that predicted by West et al. [24]: b = 2.88 (models 1 and 5), and b = 2.78 (model 9).

#### Including species as random effect improved model fit

Our study showed that including species as random effect improved model fit, which is consistent with the study of Njana et al. [61] showing that individual-tree AGB multi-species models can be improved when a species random effect is added. In forest science, mixed-effect models that consider plot as random effect include diameter growth models [62,63], height-diameter models [64–66], crown width models [67], and biomass allometric models [68,69]. Other biomass model studies have considered different variables as random effect, such as author (categorical variable encompassing differences such as methodology) [70]; tree origin (planted or natural forest) and geographic region [71]; plant family, wood density (categorical variable) and ecoregion [72]; and tree species [61].

Biomass allometric model development often results in hierarchical data grouped by plot or site and species. Same-species and same-site observations are likely to be more correlated and hence lack independence. It is important that the structure of the data is taken into account. Therefore, for this type of data, mixed-effect models should be used instead of fixed-effect models [61].

Cerrado has the highest biodiversity of any savanna in the world. Cerrado *latu sensu*, which ranges from grasslands to closed woodlands, contains 951 woody species [73], and tree biodiversity in cerrado ss is also high (50–80 species ha^-1^) [74]. However, the vegetation often consists of a few oligarchic species and a large number of rare species [73]. Thus, multi-species models are more appropriate to estimate biomass in this biome. Although it may be unrealistic to use species-specific models for species-rich forests, including the species random effect may account for variability across multiple species. Furthermore, the species random effect may also serve as proxy for species wood density (as a categorical variable).

#### Explanatory variables for individual-tree aboveground biomass

Our data showed that, in the absence of other variables, diameter (measured at 30 cm above ground) or basal area alone are good predictors of individual-tree AGB in cerrado ss. Diameter is the most significant explanatory variable in AGB models and is used as the sole variable in many models [26]. In dense tropical forests, height can be difficult to measure; however, in open woodlands, such as cerrado ss, measuring height is easier. The importance of including height in biomass allometric models has been widely discussed [52,61,75,76]. Wood density has also been considered a fundamental variable for predicting AGB [60,76,77,78]. In our study, including height by using *v* as an explanatory variable significantly improved predictions, whereas including wood density did not. In studies evaluating explanatory variables for predicting AGB in African miombo woodlands (similar to cerrado ss), some researchers observed little prediction improvement when adding height to diameter-based models [79,80], whereas others, as in the present study, found that height but not wood density significantly improved predictions [81].

#### Generalized models and regional models

Destructive sampling (measuring, harvesting, and weighing trees) is an onerous task that imposes a challenge for developing local and regional models and for large sample sizes. However, in the absence of locally developed models, generic models may be used. One example is the generic pantropical model developed by Chave et al. [4], which is based on a global database of 58 sites across a wide range of vegetation types, comprising a set of 4004 harvested trees. Generic models can provide valuable information but may introduce bias for estimates in ecosystems not represented in the dataset used to develop the models [72]. We used our destructive sampling data to compare the two models with the strongest fit (models 11 and 12), in their power-law forms, with the pantropical model from Chave et al. [4] and five regional models: three from cerrado ss sites [16,18,20], one from a campo cerrado site (open woodland) [17], and one from cerrado ss and campo cerrado sites [19] (Table 7).

**Table 7:** Comparison of tree aboveground biomass models, based on destructive sampling data of the present study.

Model	*σ* (g)	CV (%)	Reference
Model 11: *y*(*g*) = [409.047 · (*v*)^0.976^] · 1.17	3,728	73.6	Present study
*y*(*kg*) = 0.0673 · (*ρd*^2^*h*)^0.976^	3,819	75.4	[4]
Model 12: *y*(*g*) = [0.979 · (*ρv*)^0.963^] · 1.17	3,889	76.8	Present study
*y*(*g*) = 28.77 · (*d*^2^*h*)	3,921	77.4	[17]
*y*(*kg*) = −0.49129 + 0.02912 · (*d*^2^*h*)	4,002	79.0	[18]
*y*(*t*) = *e*^−10234+2.459·*ln*(*d*)+0.4111·*ln*(*h*)^	7,222	142.6	[19]
*y*(*g*) = *e*^0.6997·*ln*(*v*)+2.587^	9,289	183.4	[16]
*y*(*kg*) = *e*^−3.352+2.985·*ln*(*d*)+1.186·*ln*(*ρ*)^ · 1.071	10,533	207.9	[20]

σ = standard error, CV = coefficient of variation, *y* = tree aboveground biomass, *d* = diameter (cm) (measured at 1.30 m for models in Chave et al. [4], Ribeiro et al. [20], and Scolforo et al. [19], and measured at 30 cm in Rezende et al. [18], Delitti et al. [17], and in our study), *h* = height (m), *v* = volume (dm^3^), *ρ* = wood density (g cm^-3^ for models in Chave et al. [4] and Ribeiro et al. [20], and g dm^3^ for model 12 in our study).

The generic pantropical model data set [4] did not include cerrado ss vegetation and used diameter at breast height (dbh) as an explanatory variable, instead diameter at 30 cm above ground, as recommended for savanna woodlands. Nonetheless, the predictive performance of the pantropical model was similar that of model 11 and outperformed model 12 and the other regional models (Table 7). This result supports the idea that, in the absence of reliable local models, generic models can be useful.

#### Tree aboveground plot biomass density models

Plot *ba* can be a good predictor of tree aboveground plot biomass density, as demonstrated by the high R^2^m and low CV of our plot biomass density models. These models can be useful for large-scale biomass estimates, since individual-tree data sets are rare in the literature. Ribeiro et al. [20] also developed a model to estimate biomass density from plot *ba*. However, unlike our models, which were based on a large sample (893 plots from 77 sites), their model was based on a small sample (10 plots from a single site), which may limit its applicability.

Models 13 and 14 had the same explanatory variable (plot *ba*), but the response variables (plot biomass) were calculated differently. In model 13, plot biomass was estimated from model 11 (based on *v*), which had the strongest fit. In model 14, plot biomass was estimated from model 10 (based on *ba*). The better performance of model 14 can be explained by the fact that it did not account for the height variability of the data.

### Tree aboveground biomass density variation of cerrado *sensu stricto* and environmental influences

Tree AGB density variation in cerrado ss was high between ecoregions (8.8 to 42.2 tons ha^-1^), between sites in the same ecoregion (4.8 to 39.5 tons ha^-1^), and within sites (24.3 to 69.9 tons ha^-1^). This variation reflects the local and regional environmental heterogeneity in Cerrado. Within-site variation may be due to local physiographic heterogeneity (e.g. drainage, topography, soils), as well as local differences in disturbance regimes, including fire and harvest. High local variation imposes a significant challenge for large-scale biomass estimates that do not consider disturbance regimes and vegetation dynamics. These limitations could be overcome by regular airborne or satellite monitoring and understanding of ecological processes. Therefore, large-scale estimates should integrate all of these approaches.

When examined separately with linear regression, ecoregion, sand content, and clay content explained 42%, 11.5%, and 7.4% of AGB variation, respectively. Higher sand content in soil is associated with lower water retention. Because seasonal drought is a limiting factor for vegetation growth in the Cerrado, one would expect that higher sand content would be associated with lower AGB. However, the correlation coefficient for sand was positive. A possible reason for this finding is that many of the sites with high sand content are closer to the Amazon, where higher annual precipitation and less drought may increase AGB density. In addition, cerrado ss trees often have very deep roots that can access groundwater tables even during the drought season [82]. Therefore, soil water retention would have a stronger effect on plants with shorter root systems.

#### Ecoregion

The concept of ecoregion has long been used in biodiversity conservation [9,57,58], and more recently to estimate primary productivity and carbon balance [83] and to develop height-diameter allometric models [84–88] and biomass models [72]. Despite high variation within sites and between nearby sites in our study, ecoregion explained 42% of AGB density variation. This shows its strong potential as a parameter for classifying regional biomass variation in the Cerrado. Furthermore, including ecoregion as a random effect may improve models based on data sets collected over large spatial scales. Ecoregion is a valuable categorical variable because it integrates numerous ecological and climatic factors that likely affect AGB [72].

This study represents the largest effort to date to organize and analyze decades of biomass surveys in the Brazilian Cerrado. The region is losing natural vegetation cover at an accelerated pace, with critical consequences for climate change, biodiversity conservation, and ecosystem functions (e.g. changes in the hydrological cycle). Our findings highlight the relevance of data integration, different monitoring approaches, and an understanding of the processes and patterns that determine biomass variations at different scales.

## Supporting information

S1 Table Destructive sampling data used to develop tree aboveground allometric models for cerrado *sensu stricto* in Brazil. (XLSX)

S2 Table Destructive sampling data from Delitti et al. [17], used as an independent validation data set. (XLSX)

S3 Table Detailed data on 77 cerrado *sensu stricto* sites and their respective tree aboveground biomass density (calculated with model 11) and confidence interval limits (CIL). (XLSX)

S4 Table Plot data of the 77 cerrado sensu stricto sites in Brazil used in our analyses. For more information on site data, refer to S3 Table.
(XLSX)

S5 Table Tree aboveground biomass density of 77 cerrado *sensu stricto* sites in Brazil. Bold values indicate significant differences in mean biomass between sites (P < 0.05; Kruskal–Wallis test).
(XLSX)

S1 File Supplementary Figures. (DOCX)
