# Supplementary material for: Optimizing biomass estimates of savanna woodland at different spatial scales in the Brazilian Cerrado: Re-evaluating allometric equations and environmental influences
Source: PLoS One. 2018 Aug 1;13(8):e0196742. doi: 10.1371/journal.pone.0196742 (PMC6070178; doi:10.1371/journal.pone.0196742)
Supplement: S1 File — (DOCX) [file pone.0196742.s006.docx]

Figure A. **Homoscedasticity and normality of errors of model 1 to estimate individual-tree aboveground biomass of cerrado *sensu stricto*.**

Figure B. **Homoscedasticity and normality of errors of model 2 to estimate individual-tree aboveground biomass of cerrado *sensu stricto*.**

Figure C. **Heteroscedasticity and non-normality of errors of model 3 to estimate individual-tree aboveground biomass of cerrado *sensu stricto*.**

Figure D. **Heteroscedasticity and non-normality of errors of model 4 to estimate individual-tree aboveground biomass of cerrado *sensu stricto*.**

Figure E. **Homoscedasticity and normality of errors of model 5 to estimate individual-tree aboveground biomass of cerrado *sensu stricto*.**

Figure F. **Homoscedasticity and normality of errors of model 6 to estimate individual-tree aboveground biomass of cerrado *sensu stricto*.**

Figure G. **Homoscedasticity and normality of errors of model 7 to estimate individual-tree aboveground biomass of cerrado *sensu stricto*.**

Figure H. **Homoscedasticity and normality of errors of model 8 to estimate individual-tree aboveground biomass of cerrado *sensu stricto*.**

Figure I. **Homoscedasticity and normality of errors of model 9 to estimate individual-tree aboveground biomass of cerrado *sensu stricto*.**

Figure J. **Homoscedasticity and normality of errors of model 10 to estimate individual-tree aboveground biomass of cerrado *sensu stricto*.**

Figure K. **Homoscedasticity and normality of errors of model 11 to estimate individual-tree aboveground biomass of cerrado *sensu stricto*.**

Figure L. **Homoscedasticity and normality of errors of model 12 to estimate individual-tree aboveground biomass of cerrado *sensu stricto*.**

Plot biomass class center (ton ha^-1^)

Plot biomass class center (ton ha^-1^)

Theoretical quantiles

Theoretical quantiles

A

B

Figure M. **Homoscedasticity and normality of errors of model 13 to estimate tree aboveground plot biomass density of cerrado *sensu stricto*.**

Figure N. **Homoscedasticity and normality of errors of model 14 to estimate tree aboveground plot biomass density of cerrado *sensu stricto*.**

Figure O. **Homoscedasticity and normality of errors of model 15 to investigate environmental influences over tree aboveground biomass density of cerrado *sensu stricto* in Cerrado, Brazil.**

Figure P. **Homoscedasticity and normality of errors of model 16 to investigate environmental influences over tree aboveground biomass density of cerrado *sensu stricto* in Cerrado, Brazil.**

 Figure Q. **Homoscedasticity and normality of errors of model 17 to investigate environmental influences over tree aboveground biomass density of cerrado *sensu stricto* in Cerrado, Brazil.**

Figure R. **Homoscedasticity and normality of errors of model 18 to investigate environmental influences over tree aboveground biomass density of cerrado *sensu stricto* in Cerrado, Brazil.**

Figure S. **Homoscedasticity and normality of errors of model 19 to investigate environmental influences over tree aboveground biomass density of cerrado *sensu stricto* in Cerrado, Brazil.**


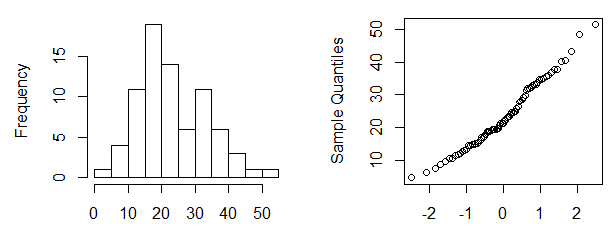


Site biomass class center (ton ha^-1^) Theoretical Quantiles

Figure T. **Histogram and QQ-Norm plot of tree-aboveground biomass density of 77 cerrado *sensu stricto* sites in Brazil, estimated using model 11.**

Figure U. **Boxplot of tree aboveground biomass density of 77 cerrado *sensu stricto* 77 sites in Brazil, estimated with model 11.**

Figure V**. Cluster analysis dendrogram for tree aboveground biomass density 77 cerrado *sensu stricto* sites in Brazil, estimated with model 11.**
